# Altered presynaptic function and number of mitochondria in the medial prefrontal cortex of adult *Cyfip2* heterozygous mice

**DOI:** 10.1186/s13041-020-00668-4

**Published:** 2020-09-11

**Authors:** Gyu Hyun Kim, Yinhua Zhang, Hyae Rim Kang, Seung-Hyun Lee, Jiwon Shin, Chan Hee Lee, Hyojin Kang, Ruiying Ma, Chunmei Jin, Yoonhee Kim, Su Yeon Kim, Seok-Kyu Kwon, Se-Young Choi, Kea Joo Lee, Kihoon Han

**Affiliations:** 1 Neural Circuits Research Group, Korea Brain Research Institute, 61, Cheomdan-ro, Dong-gu, Daegu, 41062 South Korea; 2 Department of Neuroscience, College of Medicine, Korea University, 73, Goryeodae-ro, Seongbuk-gu, Seoul, 02841 South Korea; 3 Department of Biomedical Sciences, College of Medicine, Korea University, Seoul, South Korea; 4 Department of Physiology, Dental Research Institute, Seoul National University School of Dentistry, 101, Daehak-ro, Jongno-gu, Seoul, 03080 South Korea; 5 Division of National Supercomputing, Korea Institute of Science and Technology Information, Daejeon, South Korea; 6 Korea Institute of Science and Technology, Center for Functional Connectomics, Brain Science Institute, Seoul, 02792 South Korea; 7 Department of Brain and Cognitive Sciences, DGIST, Daegu, South Korea

**Keywords:** Presynapse, Mitochondria, Medial prefrontal cortex, Cytoplasmic FMR1-interacting protein 2

## Abstract

Variants of the cytoplasmic FMR1-interacting protein (*CYFIP*) gene family, *CYFIP1* and *CYFIP2*, are associated with numerous neurodevelopmental and neuropsychiatric disorders. According to several studies, CYFIP1 regulates the development and function of both pre- and post-synapses in neurons. Furthermore, various studies have evaluated CYFIP2 functions in the postsynaptic compartment, such as regulating dendritic spine morphology; however, no study has evaluated whether and how CYFIP2 affects presynaptic functions. To address this issue, in this study, we have focused on the presynapses of layer 5 neurons of the medial prefrontal cortex (mPFC) in adult *Cyfip2* heterozygous (*Cyfip2*^*+/−*^) mice. Electrophysiological analyses revealed an enhancement in the presynaptic short-term plasticity induced by high-frequency stimuli in *Cyfip2*^*+/−*^ neurons compared with wild-type neurons. Since presynaptic mitochondria play an important role in buffering presynaptic Ca^2+^, which is directly associated with the short-term plasticity, we analyzed presynaptic mitochondria using electron microscopic images of the mPFC. Compared with wild-type mice, the number, but not the volume or cristae density, of mitochondria in both presynaptic boutons and axonal processes in the mPFC layer 5 of *Cyfip2*^*+/−*^ mice was reduced. Consistent with an identification of mitochondrial proteins in a previously established CYFIP2 interactome, CYFIP2 was detected in a biochemically enriched mitochondrial fraction of the mouse mPFC. Collectively, these results suggest roles for CYFIP2 in regulating presynaptic functions, which may involve presynaptic mitochondrial changes.

## Main text

The two members of the cytoplasmic FMR1-interacting protein (CYFIP) family, CYFIP1 and CYFIP2, are evolutionarily highly conserved proteins involved in actin cytoskeleton dynamics and mRNA regulation in neurons [1]. Importantly, both *CYFIP1* and *CYFIP2* genes are associated with various types of brain disorders, including autism spectrum disorders, intellectual disability, schizophrenia, and epilepsy, suggesting critical roles of CYFIP1 and CYFIP2 in proper brain development and function [2, 3]. Specifically, in the synaptic compartment, CYFIP1 regulates presynaptic vesicle release [4], excitatory postsynaptic dendritic spine morphology [5], and inhibitory synaptic assembly and transmission [6]. The roles of CYFIP2 in regulating dendritic spine morphology and excitatory synaptic transmission have been investigated [7–9], but its roles in presynaptic functions are largely unknown.

**Fig. 1:**
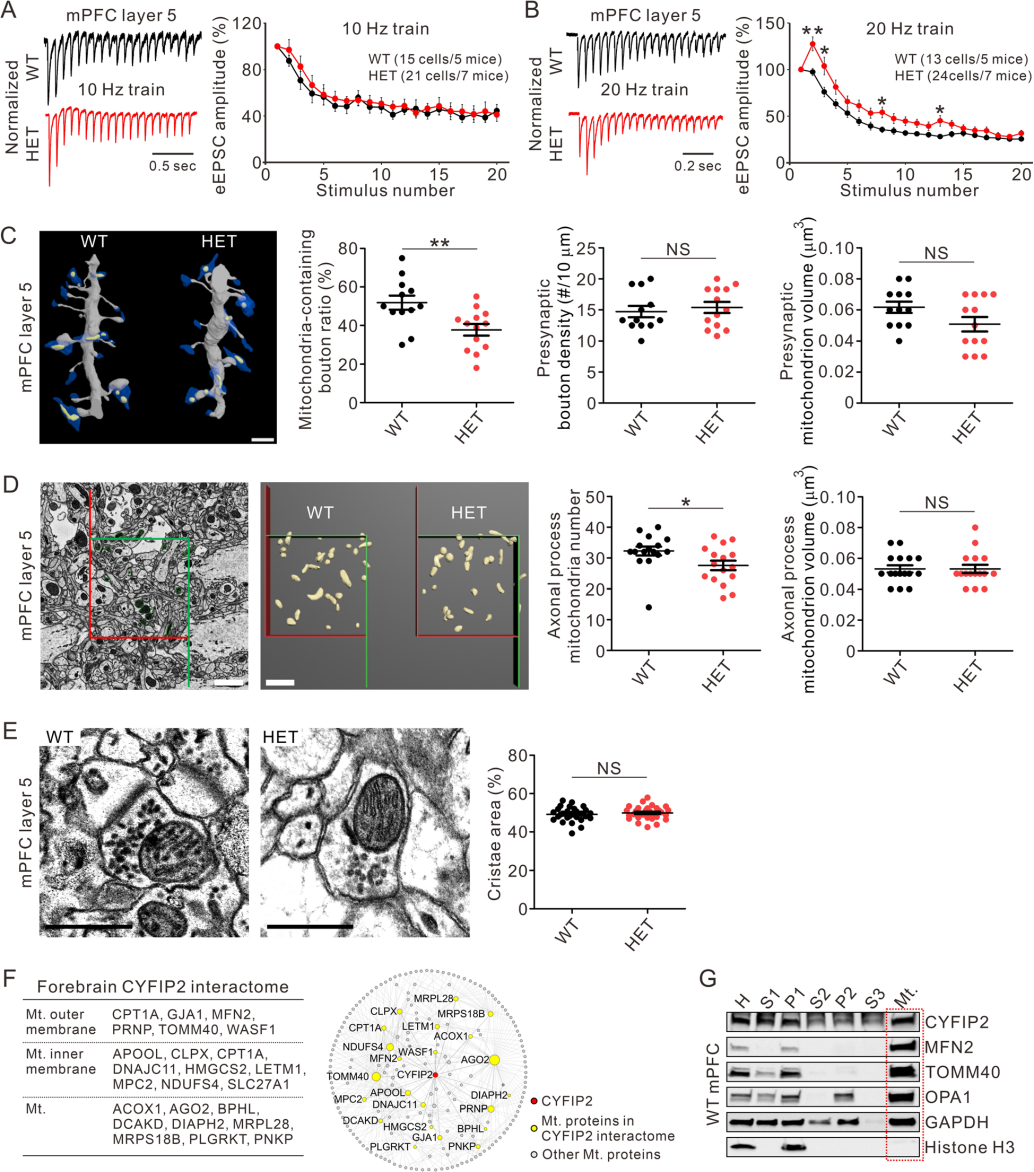
Characterization of presynaptic short-term plasticity and mitochondria in the mPFC of adult *Cyfip2*^*+/−*^ mice. **a** Representative traces and quantification of eEPSCs (normalized to the first eEPSC amplitude) induced by a 10 Hz train of stimuli in the mPFC layer 5 neurons of wild-type (WT) and *Cyfip2*^*+/−*^ (heterozygous, HET) mice. Numbers of cells and mice used for the experiments are indicated. **b** Representative traces and quantification of normalized eEPSCs induced by a 20 Hz train of stimuli. **c** Representative dendritic segments reconstructed from serial block-face scanning electron microscopy (SB-SEM) images show dendritic spines (gray), presynaptic boutons (blue), and presynaptic mitochondria (yellow) in the mPFC layer 5 neurons of WT and HET mice (left panel). Quantification of the mitochondria-containing bouton ratio, presynaptic bouton density, and presynaptic mitochondron volume (right panels, *n* = 12 and 13 dendritic segments from 4 WT and 4 HET mice, respectively). Scale bar, 2 μm. NS, not significant. **d** Representative electron microscopic image and reconstruction of axonal process mitochondria in the mPFC layer 5 of WT and HET mice (left panels). Quantification of the number (in 73.5 μm^3^) and volume of mitochondria (right panels, *n* = 16 frames from 4 mice per genotype). Scale bars, 2 μm. **e** Representative electron microscopic images of the cristae structures of presynaptic mitochondria in the mPFC layer 5 of WT and HET mice (left panels). Quantification of the mitochondrial cristae density (right panel, *n* = 30 mitochondria from 3 mice per genotype). Scale bars, 0.5 μm. **f** List of 23 mitochondrial proteins of the forebrain CYFIP2 interactome (left panel). Interaction network of 23 mitochondrial proteins (yellow nodes) in the CYFIP2 interactome. Other mitochondrial proteins (grey nodes) were also included to generate the network. Mt., mitochondria. **g** Representative western blot images showing detection of CYFIP2 in mitochondrial fraction from the mPFC homogenate of adult WT mice. Mitofusin 2 (MFN2), translocase of outer mitochondrial membrane 40 (TOMM40), and optic atrophy 1 (OPA1) are representative mitochondrial proteins. Histone H3 is a nuclear protein (negative control). Data are presented as mean ± SEM. *P < 0.05, **P < 0.01. (unpaired two-tailed Student’s *t*-test)

In our previous study, we observed a decrease in the number of presynaptic docked vesicles in layer 5 (L5) neurons in the medial prefrontal cortex (mPFC) of adult *Cyfip2* heterozygous (*Cyfip2*^*+/−*^) mice compared with wild-type mice [8]. Therefore, in this study, we further investigated the presynaptic functional changes in the mPFC L5 neurons of *Cyfip2*^*+/−*^ mice by measuring the short-term plasticity induced by trains of stimuli [see Additional File 1 for methods]. There was no significant difference between wild-type and *Cyfip2*^*+/−*^ neurons in terms of the normalized evoked excitatory postsynaptic currents (eEPSCs) induced by a moderate-frequency (10 Hz) train of stimuli (Fig. 1a). Both neurons showed similar timings of depression during the train. However, with a high-frequency (20 Hz) train of stimuli, the normalized eEPSCs were significantly different between wild-type and *Cyfip2*^*+/−*^ neurons (Fig. 1b). Specifically, *Cyfip2*^*+/−*^ neurons showed an overall enhancement in normalized eEPSCs compared with wild-type neurons, suggesting altered short-term plasticity induced by high-frequency stimulation in *Cyfip2*^*+/−*^ neurons.

Presynaptic Ca^2+^ is critically involved in short-term plasticity [10], and presynaptic mitochondria play an important role in buffering presynaptic Ca^2+^ [11, 12]. Notably, a recent study has revealed changes in mitochondrial activity and size in *Drosophila Cyfip* mutants [13]. Therefore, we also investigated the number and morphology of presynaptic mitochondria in the mPFC L5 neurons of *Cyfip2*^*+/−*^ mice by re-analyzing electron microscopic image datasets previously acquired for dendritic spine analysis of the mPFC neurons [8] [see Additional File 1 for methods]. We found that mitochondria were observed only in a subpopulation of presynaptic boutons contacting dendritic spines of the mPFC L5 neurons, and that the ratio of presynaptic boutons containing mitochondria in *Cyfip2*^*+/−*^ mice was significantly lower than that observed in wild-type mice (Fig. 1c). Considering normal densities of dendritic spines [8] and presynaptic boutons (Fig. 1c) of mPFC L5 neurons in *Cyfip2*^*+/−*^ mice, these results indicate that *Cyfip2*^*+/−*^ neurons have more mitochondria-free presynaptic boutons. Additionally, mitochondria number in axonal processes of the mPFC L5 was also significantly decreased in *Cyfip2*^*+/−*^ mice compared with wild-type mice (Fig. 1d), suggesting that mitochondria transport along the axons can also be abnormal in *Cyfip2*^*+/−*^ mice. In contrast, the mitochondrial volume in neither presynaptic boutons nor axonal processes was altered in *Cyfip2*^*+/−*^ mice (Fig. 1c,d). As mitochondrial cristae density is highly correlated with its energy production and metabolic capacity [14], we next analyzed the mitochondrial cristae density in *Cyfip2*^*+/−*^ and wild-type mice. Consistent with no difference in presynaptic mitochondrion volume (Fig. 1c), the cristae density of presynaptic mitochondria was comparable between genotypes (Fig. 1e). These results suggest that *Cyfip2* haploinsufficiency affects the axonal and presynaptic localization, rather than the volume or cristae integrity, of mitochondria in the mPFC layer 5.

We have recently identified 140 proteins in the CYFIP2 interactome of mouse forebrain, which mainly included actin-regulatory proteins and RNA-binding proteins [15]. Notably, we found that 23 mitochondrial proteins were also detected in the CYFIP2 interactome (Fig. 1f). To further validate whether CYFIP2 was associated with mitochondria in the mPFC, we biochemically enriched the mitochondrial fraction from the mPFC homogenate of adult wild-type mice [see Additional File 1 for methods]. Western blot analysis showed that CYFIP2 was detected in the mPFC mitochondrial fraction together with other mitochondrial proteins (Fig. 1g).

In this study, we showed changes in presynaptic short-term plasticity in the mPFC L5 neurons of adult *Cyfip2*^*+/−*^ mice. The enhancement in the normalized eEPSCs in *Cyfip2*^*+/−*^ neurons was induced by high-frequency, but not low-frequency, stimuli, suggesting that the decay of presynaptic Ca^2+^ signaling, such as Ca^2+^ clearance, rather than the action potential-induced Ca^2+^ increase, may be altered in *Cyfip2*^*+/−*^ neurons. Presynaptic mitochondria positively regulate Ca^2+^ clearance in the axon terminal and thereby modulate neurotransmitter release [12]. Therefore, we speculate that the reduced number of presynaptic mitochondria in *Cyfip2*^*+/−*^ neurons may decrease presynaptic Ca^2+^ clearance, which in turn elevates the residual Ca^2+^, and may trigger the short-term facilitating transmission. However, other possible mechanisms should also be considered. For example, mitochondria generate the majority of presynaptic ATP via oxidative phosphorylation, which is critical for synaptic vesicle recycling [11]. Therefore, changes in presynaptic ATP levels owing to the reduced mitochondria number may also contribute to the abnormal short-term plasticity in *Cyfip2*^*+/−*^ neurons. In addition, mitochondria-independent mechanisms, such as changes in presynaptic actin dynamics, can be involved in the presynaptic functional changes of *Cyfip2*^*+/−*^ neurons, as previously shown in *Cyfip1*^*+/−*^ neurons [4]. Furthermore, in our previous study [8], we showed that the number of docked vesicles in a single presynaptic terminal was lower in *Cyfip2*^*+/−*^ neurons compared with wild-type neurons, which is also in agreement with our current finding of an enhancement in the presynaptic short-term plasticity and an increase in the pared pulse ratio in *Cyfip2*^*+/−*^ neurons.

Our observations of reduced number, but normal volume, of presynaptic mitochondria in *Cyfip2*^*+/−*^ neurons contradict the results of a recent report showing increased mitochondrial activity and size in *Drosophila Cyfip* mutants [13]. Only one *Cyfip* gene exists in *Drosophila*, but there are two genes, *Cyfip1* and *Cyfip2*, in mice. Therefore, one possibility can be that CYFIP1 and CYFIP2 have different roles in regulating presynaptic mitochondria in the mouse brain. Our recent interactome analysis suggested that CYFIP1 and CYFIP2 have distinct pools of binding partners [15], supporting their differential molecular functions in vivo.

Detailed mechanisms underlying the reduced number of mitochondria in presynaptic boutons and axonal processes of *Cyfip2*^*+/−*^ mPFC require further investigation. Presynaptic mitochondrial transport and localization can be regulated by several mechanisms, including the Ca^2+^-dependent detachment of the mitochondria from the molecular motor and its immobilization by mitochondrial docking proteins [16]. Whether CYFIP2 is involved in mitochondrial capture at presynaptic sites via interactions with mitochondrial proteins will be an interesting topic of future studies.

Notably, there are several intra-mitochondrial and mitochondrial inner membrane proteins in the CYFIP2 interactome (Fig. 1f). However, we could not find CYFIP2 in the publicly available mitochondrial protein databases (MitoCarta [17] and MitoProteome [18]). Furthermore, intra-mitochondrial localization was not predicted for CYFIP2 by a prediction tool of protein sub-mitochondrial localization (DeepMito [19]). These results suggest that CYFIP2 is not an intra-mitochondrial protein, and that those interactions between CYFIP2 and intra-mitochondrial proteins can be indirect via mitochondrial outer membrane proteins. Further investigations are necessary to identify key mitochondrial proteins mediating CYFIP2-mitochondira interaction and its potential regulation.

In conclusion, our results provide in vivo evidence that CYFIP2 regulates presynaptic functions, which may involve presynaptic mitochondrial changes. These results may be potentially implicated for *CYFIP2*-associated brain disorders, given that presynaptic mitochondrial dysfunction contributes to the pathogenesis of various brain disorders [11].

## Supplementary information

**Additional file 1.**

## Data Availability

The datasets used and analyzed in the current study are available from the corresponding authors on reasonable request.

## Abbreviations

CYFIP: Cytoplasmic FMR1-interacting protein
eEPSC: Evoked excitatory postsynaptic current
HET: Heterozygous
L5: Layer 5
MFN2: Mitofusin 2
mPFC: Medial prefrontal cortex
OPA1: Optic atrophy 1
SB-SEM: Serial block-face scanning electron microscopy
TOMM40: Translocase of outer mitochondrial membrane 40
WT: Wild-type
