# Supplementary material for: Altered presynaptic function and number of mitochondria in the medial prefrontal cortex of adult Cyfip2 heterozygous mice
Source: Mol Brain. 2020 Sep 11;13:123. doi: 10.1186/s13041-020-00668-4 (PMC7488858; doi:10.1186/s13041-020-00668-4)
Supplement: Supplementary file 1 — Additional file 1. [file 13041_2020_668_MOESM1_ESM.docx]

**Additional File 1.**

**Altered presynaptic function and number of mitochondria in the medial prefrontal cortex of adult *Cyfip2* heterozygous mice**

Gyu Hyun Kim, Yinhua Zhang, Hyae Rim Kang, Seung-Hyun Lee, Jiwon Shin, Chan Hee Lee, Hyojin Kang, Ruiying Ma, Chunmei Jin, Yoonhee Kim, Su Yeon Kim, Seok-Kyu Kwon, Se-Young Choi, Kea Joo Lee, and Kihoon Han

**Materials and Methods**

**Mice**

The *Cyfip2^+/-^* mice used in this study have been previously described [1, 2]. The mice were bred and maintained in a C57BL/6J background according to the Korea University College of Medicine Research Requirements, and all the experimental procedures were approved by the Committees on Animal Research at the Korea University College of Medicine (KOREA-2018-0174). The mice were fed *ad libitum* and housed under a 12 h light-dark cycle. All experiments were performed with adult (6 to 10-week-old) male mice.

**Electrophysiology**

Coronal slices (300 μm thickness) containing the medial prefrontal cortex (mPFC) were prepared from the brains of the isoflurane-anesthetized mice as described previously [2]. The slices were kept in ice-cold, oxygenated (95% O_2_/5% CO_2_), low-Ca^2+^/high-Mg^2+^ dissection buffer containing (in mM) 5 KCl, 1.23 NaH_2_PO_4_, 26 NaHCO_3_, 10 dextrose, 0.5 CaCl_2_, 10 MgCl_2_, and 212.7 sucrose, and then transferred to a storage chamber in an incubator containing oxygenated artificial cerebrospinal fluid (ACSF) containing (in mM) 124 NaCl, 2.5 KCl, 1.23 NaH2PO4, 2.5 CaCl_2_, 1.5 MgCl_2_, 26 NaHCO_3_, and 10 dextrose at 28–30°C for at least 30 min before recording. Visualized whole-cell recordings were made with an infrared-differential interference contrast microscope (BX51WI; Olympus). Slices were transferred to a recording chamber where they were perfused with oxygenated ACSF (23–25°C) at a flow rate of 2 mL/min. Synaptic responses were recorded in layer 5 and evoked with 0.2 ms current pulses delivered with a bipolar stimulating electrode (200 μm diameter; FHC, Bowdoinham, ME) placed approximately in layer 2 at intensities that produced 40–50% of the maximal excitatory postsynaptic current (EPSC) amplitude. Patch pipettes (4–6 MΩ) were filled with a solution containing (in mM) 130 Cs-MeSO_4_, 0.5 EGTA, 5 TEA-Cl, 8 NaCl, 10 HEPES, 1 QX-314, 4 ATP-Mg, 0.4 GTP-Na, 10 phosphocreatine-Na_2_, 0.1 spermine, pH 7.4, 275–285 mOsm. The extracellular recording solution consisted of ACSF supplemented with picrotoxin (100 μM). Data were acquired using an EPC-8 amplifier (HEKA), filtered at 3 kHz, digitized at 10 kHz with Digidata 1550B (Axon Instruments), and analyzed using pClamp 10 (Molecular Devices). Only cells with access resistance <20 MΩ and input resistance >100 MΩ were studied. Cells were discarded if the input or access resistance changed by more than 20%.

**Electron microscopy**

The mPFC of adult *Cyfip2^+/-^* and wild-type mice (n = 4 per group) was used to produce serial block-face scanning electron microscopy (SB-SEM) datasets. Briefly, mice were perfused with 2% paraformaldehyde and 2.5% glutaraldehyde in 0.15 M cacodylate buffer (pH 7.4). The brains were sliced into 150 μm coronal sections and dissected into the prelimbic mPFC. The slices were postfixed in 2% OsO_4_/1.5% potassium ferrocyanide for 1 h. Samples were then immersed in 1% thiocarbohydrazide (Ted Pella) in ddH2O for 20 min followed by 2% OsO_4_ for 30 min. Tissues were incubated in 1% uranyl acetate at 4˚C overnight and lead aspartate solution at 60˚C for 30 min. Specimens were dehydrated using a series of ethanol followed by acetone for 10 min. Tissues were embedded in 7% (w/v) conductive Epon 812 resin (EMS) mixed with Ketjen black powder [3]. Tissue blocks were imaged with a Merlin VP field-emission SEM (Carl Zeiss) equipped with 3View2 (Gatan). Serial images were acquired with a 30 μm aperture, high vacuum, voltage of 2.5 kV, image size of 5,000 x 5,000 pixels, and X-Y resolution of 12 nm at a nominal thickness of 50 nm. A stack of 210 serial images were obtained per mouse (37,800 μm^3^ per stack), in layer 5 of the prelimbic mPFC. Images were processed with ImageJ/Fiji plugins (http:// http://fiji.sc/Fiji). TrakEM2 was used to align images for 3D reconstruction. We randomly selected spiny dendritic segments with lengths of at least 12 μm. To ensure that our analysis was restricted to pyramidal neurons, we avoided all dendritic segments with few or no spines. In total, 25 dendritic branches including all the protrusions and their corresponding presynaptic boutons were manually segmented in the mPFC layer 5 (12 wild-type and 13 *Cyfip2^+/-^*) using the reconstruct software (https://synapseweb.clm.utexas.edu/software-0) by three annotators blinded to the genotypes. The ratio of presynaptic boutons containing mitochondria and the volume of mitochondria at the presynaptic site were measured to compare differences between groups. In addition, we applied an established physical disector principle to measure the overall density of mitochondria in the axonal processes. In four sampling areas of 30 serial images per animal, the number of presynaptic axonal mitochondria within each image stack was divided by the disector volume (73.5 μm^3^ each) to produce the overall density of presynaptic axonal mitochondria (n = 16 stacks from 4 animals per group, total disector volume of 1,176 μm^3^ per group). The volume of individual mitochondria in presynaptic axonal processes was also measured using the reconstruct software. For the cristae density, the mitochondrial cristae area was divided by total mitochondrion area (n = 30 mitochondria from 3 animals per group).

**Biochemistry and western blot analysis**

To obtain crude mitochondrial fraction, we slightly modified the protocol from Wieckowski et al. [4]. Mouse brain tissue was washed four times with 2 mL ice-cold Buffer C (225 mM D-mannitol, 75 mM sucrose, 30 mM Tris-HCl, pH7.4) to remove the blood. Then, the tissue was washed once again with 2 mL of ice-cold Buffer A (225 mM D-mannitol, 75 mM sucrose, 30 mM Tris-HCl, pH7.4, 0.5% BSA, 0.5 mM EGTA). Tissue was homogenized with Buffer A containing freshly added protease and phosphatase inhibitors in a ratio of 4 mL buffer per gram of tissue. Homogenate was centrifuged at 1,000 g for 5 min at 4°C (the pellet after this step is the P1 fraction containing unbroken cells and nuclei) and the supernatant was centrifuged again at 1,000 g for 5 min at 4°C (the supernatant after this step is the S1 fraction). The supernatant containing crude mitochondrial fraction was transferred to a new tube and centrifuged at 9,000 g for 10 min at 4°C. Supernatant after this centrifuge was the S2 fraction containing lysosomes and microsomes. The pellet was gently resuspended in 500 μL of ice-cold Buffer A and centrifuged at 9,000 g for 10 min at 4°C. To release synaptic mitochondria, the pellet was resuspended in 200 μL of ice-cold Buffer B (225 mM D-mannitol, 75 mM sucrose, 30 mM Tris-HCl, pH7.4, 0.5% BSA, the fraction after resuspension was P2), and frozen and thawed three times with liquid nitrogen. The mitochondrial suspension was centrifuged at 10,000 g for 10 min at 4°C. The crude mitochondrial pellet was resuspended in 1 mL of Buffer C and centrifuged again at 10,000 g for 10 min at 4°C. Finally, the crude mitochondrial pellet was resuspended in 100 μL of ice-cold Resuspension Buffer (250 mM D-mannitol, 5 mM HEPES, pH 7.4, 0.5 mM EGTA) with freshly added protease and phosphatase inhibitors. All buffers were prepared freshly right before use. Antibodies used for western blot analysis were CYFIP2 (Abcam, ab95969), GAPDH (Cell Signaling, #2118), Histone H3 (Cell Signaling, #4499), MFN2 (Abcam, ab56889), OPA1 (BD Biosciences, #612606), and TOMM40 (Proteintech, 18409-1-AP).

**References**

1. Han K, Chen H, Gennarino VA, Richman R, Lu HC, Zoghbi HY. Fragile X-like behaviors and abnormal cortical dendritic spines in Cytoplasmic FMR1-interacting protein 2-mutant mice. Human molecular genetics. 2015;24(7):1813-23. doi:10.1093/hmg/ddu595.

2. Lee SH, Zhang Y, Park J, Kim B, Kim Y, Lee SH et al. Haploinsufficiency of Cyfip2 Causes Lithium-Responsive Prefrontal Dysfunction. Ann Neurol. 2020. doi:10.1002/ana.25827.

3. Nguyen HB, Thai TQ, Saitoh S, Wu B, Saitoh Y, Shimo S et al. Conductive resins improve charging and resolution of acquired images in electron microscopic volume imaging. Sci Rep. 2016;6:23721. doi:10.1038/srep23721.

4. Wieckowski MR, Giorgi C, Lebiedzinska M, Duszynski J, Pinton P. Isolation of mitochondria-associated membranes and mitochondria from animal tissues and cells. Nat Protoc. 2009;4(11):1582-90. doi:10.1038/nprot.2009.151.
